# A symptomatic approach to tuberculosis screening for high-risk groups in Malaysia: Cost-effectiveness and budget impact analysis

**DOI:** 10.1016/j.jctube.2022.100334

**Published:** 2022-10-12

**Authors:** Nor Zam Azihan Mohd Hassan, Asmah Razali, Mohd Shaiful Jefri Mohd Nor Sham Kunusagaran, Farhana Aminuddin

**Affiliations:** a Center for Health Economics Research, Institute for Health Systems Research (IHSR), Ministry of Health Malaysia, Shah Alam, Selangor Darul Ehsan, Malaysia; b Disease Control Division, Ministry of Health Malaysia, Wilayah Persekutuan Putrajaya, Malaysia

**Keywords:** Cost-Effectiveness Analysis, Budget Impact Analysis, Tuberculosis, Screening, Health

## Abstract

**Background:**

One of the crucial areas of eliminating TB is the early detection of cases through the screening programme. This study's focal point is to measure the relative cost-effectiveness and budget impact of the symptomatic approach compared to the existing approach of TB screening (consisted of both symptomatic and asymptomatic screening) among high-risk groups in Malaysia.

**Method:**

The Cost-Effectiveness Analysis (CEA) was measured using a decision tree model and the outcome is presented in terms of cost per TB case detected and the ICER. Analysis was conducted using secondary data collected from the Disease Control Division, Sabah and Sarawak State Health Departments. The robustness of the model is determined by Deterministic and Probabilistic Sensitivity Analysis. Subsequently, Budget impact over 5 years period, from 2018 to 2022 was estimated for both approaches.

**Results:**

The symptomatic approach was more cost-effective than the existing approach with ICER of MYR 424.71. The cost of conducting Sputum for Acid Fast Bacilli (SAFB) was found as the key driver for ICER. Budget Impact Analysis showed that switching from the existing approach towards the symptomatic approach would result in a cost-saving of MYR 65.5 million over 5 years. However, this would lead to 4473 TB cases being undetected over the same duration.

**Conclusion:**

This study suggests that the policymakers have to weigh the trade-off between cost-saving and TB case detection. This cost-saving can be channelled to new TB intervention programme with a higher chance of TB case detection. Nevertheless, any decision made must be in concordance to the objectives of the country’s TB programme.

## Highlights

• Symptomatic Approach for TB screening among high-risk groups was more cost-effective than the existing approach.
• Cost of conducting Sputum Acid Fast Bacilli (SAFB) was the key driver in determining which approach is more cost-effective.
• Switching from existing approach to symptomatic approach would results in cost-savings of MYR 65.5 million, but resulted in 4473 TB cases undetected in 5 years period.

## 1 Introduction

Tuberculosis (TB) is caused by Mycobacterium tuberculosis, and one of the oldest infectious diseases known to infect the human [1]. It remains one of the major public health challenges especially in the low- and middle-income countries despite the advancement in treatment and management. Recent data shows that it has persistently killed millions of people annually since it was first recorded in the 1960s and has since ranked as one of the leading causes of morbidity and mortality by a single infectious agent [2]. In 2019, around 10.0 million people developed TB, of which 1.2 million TB deaths occurred among HIV-negative people and 208,000 deaths among people living with HIV (PL HIV) [3]. From the total global TB burden, more than half of the infections occur in the Western Pacific region.

Ending TB epidemic was set as one of the Sustainable Development Goals (SDGs) target to be achieved by 2030 [4]. In reaction to this, World Health Organization (WHO) through the WHO End TB Strategy has urged for reduction of TB deaths and TB incidence rate by 95 % and 90 % respectively, by 2035 [4], [5]. Pillar 1 of WHO End TB Strategy, of which to provide integrated, patient-centred care and prevention, focus must be given to early detection, treatment and prevention to all. Hence, by ensuring equal access to healthcare and better engagement with the patient as well as community. There is no “one size fits all” approach in managing TB, therefore, each country has to adapt based on their diverse country setting.

While TB can infect every-one, certain groups of population such as PL HIV, healthcare workers, and those living in institutional setting have higher risk of contracting with TB infection. TB infection occur upon exposure to an infectious individual, spreading through an airborne. While TB can affect various body sites, TB is primarily affecting the lungs [6]. Hence, the mainstay of TB detection focuses on Sputum for Acid Fast Bacilli (SAFB) and Chest X-ray (CXR). There are continuous debates on various TB screening approaches due to the presence of asymptomatic cases [6]. Generally, TB screening can be done either through targeted screening of high-risk groups or mass population screening [7]. Nevertheless, studies have shown that the high-risk groups have higher TB incidence than the general population [8], [9]. Hence, WHO has suggested for a more systematic approach to TB screening compared to the mass population screening. Even though symptom screening was shown to have sensitivity around 69 %, the addition of CXR would results in around 2.5-fold increase in TB case detection and an increase the sensitivity as much as 11 % [10].

Malaysia has achieved quite remarkably in terms of controlling TB infection. Malaysia TB notification rate has remained below 100 cases per 100,000 population for the past few years, however, it is still one of the leading causes of morbidity and mortality among infectious diseases [2]. Nevertheless, TB infections still posed as major threats in institutionalized setting especially in prisons with incidence rates about ten times of general population. Whereas, 6.3 % of TB cases undergone HIV screening turned out as HIV-positive [11]. To achieve higher case detection rate, MOH Malaysia has introduced The Malaysian National Tuberculosis Programmes (NTPs) by expanding the prior TB control strategy, giving focus on early case detection among those having TB symptoms and high-risk groups, at the same time providing quality laboratory services, develop training modules and guidelines, conducting routine training to staffs, plus inter agency collaborations [11].

As one of the main strategies for Ministry of Health (MOH) Malaysia to control TB infection, TB screening among the high-risk groups include those close contact to TB cases, immunocompromised patients, an elderly and others involves both the asymptomatic and symptomatic [12]. According to the TB screening guideline for the high-risk groups, MOH Malaysia, TB screening method is decided upon whether the person is symptomatic or asymptomatic. A person without any symptom is subjected for CXR, while the those with TB symptoms such as cough, loss of appetite and loss of weight will require both CXR and SAFB [12]. Despite that, studies showed that TB screening among the asymptomatic individual using CXR, often yields unreliable results [13]. For example, a study among the asymptomatic in Malaysia revealed a low yield, of which PL HIV was the highest with 25 %. The lowest yield was among close contact of TB case with 4.4 % [14]. Hence, creating debates on the value of screening the asymptomatic.

To achieve the WHO goal for TB in 2035, countries must provide full commitment in terms of funding for tuberculosis care, prevention and treatment. With the budget constraints and increasing health expenditure, prioritization of TB screening strategy is necessary. This includes the decision on whether to screen only those presented with symptoms or both the asymptomatic and symptomatic high-risk groups [15]. An unsystematic and poorly targeted TB screening approach will not only add unnecessary burden to the healthcare systems, but also results in high operational cost. [15], [16]. In response, WHO has suggested for a more systematic approach to TB screening [17]. TB screening strategy should take into account the measure of cost-effectiveness while focusing on the high-risk groups [15]. At the same time, Budget Impact Analysis (BIA) should be incorporated into the Cost-Effectiveness Analysis (CEA) for better interpretation of the result, which will allow the policy makers to make better decision. Prior studies have revealed that less than 5 % of global health CEA include BIA in their study [18].

The government through MOH Malaysia has invested lots of resources to control TB infection, especially through screening of the high-risk groups, of which the approach includes screening both the asymptomatic and symptomatic. However, there are lack of documented studies that measure the economic efficacy and the long-term impact of this TB screening approach. With the increasing pressure to end TB epidemic, new programmes were introduced such as screening and treatment of latent TB as well as the introduction of the novel Xpert MTB/RIF diagnostic technology to improve TB case detection. These new and existing programmes give extra pressures to MOH in terms of budget allocations. Hence, this study focuses on measuring the cost-effectiveness and the budget impact of symptomatic approach to TB screening compared to the existing approach (screening both the asymptomatic and symptomatic) among the high-risk groups in Malaysia. This study is conducted from the health care provider perspective (MOH, Malaysia).

## 2 Methods and materials

This paper is a subset to the study on CEA of High-Risk Groups Tuberculosis Screening in Malaysia, which aimed to measure the relative cost-effectiveness and estimates the five years budget impact of symptomatic TB screening compared to the existing approach [19]. To answer the study objective, two hypotheses scenario were developed, namely existing approach and symptomatic approach. Existing approach is defined as current TB screening approach, of which both symptomatic and asymptomatic high-risk groups were screened. Symptomatic approach was the alternative hypothesis approach, of which only the symptomatic high-risk groups were screened for TB. Data were gathered from multiple sources for the analysis, including the Disease Control Division, MOH, TB Information system (TBIS 204S) as well as Sabah and Sarawak State Health Departments. First, a decision tree model was developed for CEA to compare the two scenarios. The findings from CEA were subsequently introduced into BIA. The outcome of BIA was presented in term of number of TB cases detected and total costs for both scenarios over the span of 5 years, from 2018 to 2022. Costs data were valued in year 2018 using Malaysia Ringgit (MYR). The Willingness to Pay (WTP) threshold of three times GDP per capita was used [19], [20], [21]. Based on 2018 Malaysia GDP per capita of ∼ MYR 40,000, WTP threshold was capped at MYR120,000, where MYR 1.00 ∼ USD 0.25 [22], [23].

### 2.1 Clinical data

The number of TB cases detected were identified by assessing the number of TB screening and the number of TB cases identified through TB screening procedure. A three years of TB screening data in the period of 2016 to 2018 from Sabah and Sarawak were examined to increase the reliability of the data. These data were extracted from TBIS 204S. Only 11 high-risk groups were included in the study selected based the TB screening guideline for the high-risk groups for MOH, Malaysia. Excluded were those without results, diagnosed by specialists, diagnosed by other modalities, and TB screening of close contact. From the total list provided, only 65,400 cases were included. The estimated effectiveness data are shown in Table 1.

**Table 1:** Estimated Effectiveness Data for 11 High-Risk Groups.

TB Screening for High-Risk Groups	TB Cases Detected (per 1000 Screening)	Range¶	Distributions§	Alpha	Beta
Symptomatic TB screening for high-risk groups					
COAD patients	20.4	15.3–25.5	Beta	15.65	751.66
CCRC inmates	0.0	na	na	na	na
DM patients	23.4	17.6–29.3	Beta	15.34	640.15
ESRF patients	75.3	56.5–94.1	Beta	14.76	181.25
Smokers	37.6	28.2–47.0	Beta	15.36	393.17
PL HIV	57.5	43.1–71.9	Beta	15.18	248.84
Methadone Clinic clients	0.0	na	na	na	na
Prisoners	97.3	73.0–121.6	Beta	14.38	133.37
ENH residents	0.0	na	na	na	na
RA patients	0.0	na	na	na	na
Elderly§	3.5	2.6–4.4	Beta	15.27	310.27
Asymptomatic TB screening for high-risk groups					
COAD patients	1.8	1.4–2.3	Beta	12.93	7173.11
CCRC inmates	3.3	2.5–4.1	Beta	16.96	5121.28
DM patients	1.5	1.1–1.9	Beta	14.04	9345.90
ESRF patients	1.0	0.8–1.3	Beta	11.10	11087.90
Smokers	2.4	1.8–3.0	Beta	15.96	663.71
PL HIV	6.1	4.6–7.6	Beta	16.43	2677.16
Methadone Clinic clients	0.0	na	na	na	na
Prisoners	8.4	6.3–10.5	Beta	15.86	1871.90
ENH residents	0.0	na	na	na	na
RA patients	0.0	na	na	na	na
Elderly§	46.9	35.2–58.6	Beta	15.07	4289.80

COAD Constrictive Obstructive Airway Disease, CCRC Cure and Care Rehabilitation Centre, DM Diabetes Mellitus, ESRF End Stage Renal Failure, PL HIV Person Living with Human Immunodeficiency Virus, ENH Elderly Nursing Home, RA Rheumatoid Arthritis, TB Tuberculosis, na not available.

§ Elderly (60 years and above).

¶ The data are varied by ± 25 %.

By using similar data, the probabilities data were estimated. These were the probabilities of those undergone TB screening presented with symptoms (being symptomatic) and the probabilities of each high-risk group among the symptomatic or asymptomatic cases. The probabilities data are shown in Table 2.

**Table 2:** Probabilities Data.

Probability Data	Probability Value	Range^¶^	Distributions^§^	Alpha	Beta
Probability cases of presented with symptom (symptomatic)	0.1082	0.0811–0.1352	Beta	14.19	117.07
Probability of high-risk groups among symptomatic cases:					
COAD patients	0.0416	0.0312–0.0519	Beta	15.29	352.32
CCRC inmates	0.0045	0.0034–0.0057	Beta	16.66	3684.62
DM patients	0.3987	0.2990–0.4984	Beta	9.22	13.90
ESRF patients	0.0131	0.0099–0.0164	Beta	15.54	1170.64
Smokers	0.0489	0.0367–0.0611	Beta	15.23	296.24
PL HIV	0.0123	0.0092–0.0154	Beta	15.54	1247.64
Methadone Clinic clients	0.0001	0.0001–0.0002	Beta	11.11	111087.89
Prisoners	0.0886	0.0665–0.1108	Beta	14.43	148.42
ENH residents	0.0025	0.0019–0.0032	Beta	17.32	6908.77
RA patients	0.0008	0.0006–0.0011	Beta	15.99	19967.01
Elderly^§^	0.3887	0.2915–0.4859	Beta	9.39	14.76
Probability of high-risk groups among asymptomatic cases:					
COAD patients	0.0186	0.0140–0.0233	Beta	15.35	810.00
CCRC inmates	0.0156	0.0117–0.0194	Beta	15.73	992.91
DM patients	0.5970	0.4478–0.7463	Beta	5.85	3.95
ESRF patients	0.0339	0.0255–0.0424	Beta	15.33	436.97
Smokers	0.0211	0.0158–0.0263	Beta	15.49	718.81
PL HIV	0.0057	0.0042–0.0071	Beta	16.48	2874.11
Methadone Clinic clients	0.0002	0.0001–0.0002	Beta	4.00	19991.00
Prisoners	0.1789	0.1342–0.2237	Beta	12.97	59.54
ENH residents	0.0071	0.0053–0.0089	Beta	15.44	2159.36
RA patients	0.0004	0.0003–0.0006	Beta	15.99	39967.01
Elderly^§^	0.1215	0.0911–0.1519	Beta	13.91	100.59

COAD Constrictive Obstructive Airway Disease, CCRC Cure and Care Rehabilitation Centre, DM Diabetes Mellitus, ESRF End Stage Renal Failure, PL HIV Person Living with Human Immunodeficiency Virus, ENH Elderly Nursing Home, RA Rheumatoid Arthritis, TB Tuberculosis, na not available.

§ Elderly (60 years and above).

¶ The data are varied by ± 25 %.

### 2.2 Costs

Two TB screening procedures were CXR and SAFB. The TB screening related costs from MOH perspective were calculated based on the capital (such as machineries plus their yearly maintenance and calibrations), personnel (such as yearly salary and allowances for staffs involved) and consumables (such as include the X-ray film, chemicals, personal protective equipment and others) involved. A mix of step down and Activity Based Costing (ABC) were implemented for costs estimation. Expert opinion was used to allocate the costs. The allocation of capital and personnel costs were based on duration of the respective screening procedure. The cost of screening asymptomatic case equals to the costs of one CXR procedure, while the costs of conducting TB screening among the symptomatic was the summation of both costs for CXR and SAFB. (Table 3).

**Table 3:** Cost for TB Screening.

Cost Data	Unit Cost (MYR)	Range (MYR)¶	Distributions
TB screening for asymptomatic case			
CXR cost	40.27	30.20 – 50.34	Gamma
**Total**	**40.27**		
			
TB screening for symptomatic case			
CXR cost	40.27	30.20 – 50.34	Gamma
SAFB cost	16.38	12.29 – 20.48	Gamma
**Total**	**56.65**		

CXR Chest X-ray, SAFB Sputum for Acid Fast Bacilli,

¶ The costs data are varied by ± 25 %.

### 2.3 Cost-Effectiveness

**Fig. 1:**
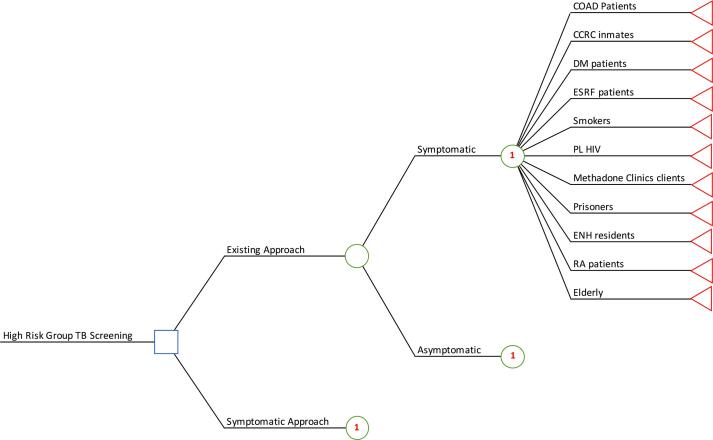
Decision Tree Model for Cost-Effectiveness Analysis. COAD Constrictive Obstructive Airway Disease, CCRC Cure and Care Rehabilitation Centre, DM Diabetes Mellitus, ESRF End Stage Renal Failure, PL HIV Person Living with Human Immunodeficiency Virus, ENH Elderly Nursing Home, RA Rheumatoid Arthritis.

The CEA was evaluated over one-year time horizon, with existing approach as the comparator. TreeAge Pro Healthcare version 2019 was used to develop a decision tree model for the CEA, of which populated with the cost, probabilities and effectiveness data (Table 2, Table 3, Table 4). The decision tree model is depicted as in Fig. 1. The symptomatic approach was compared to the existing TB screening approach. The existing approach consisted of symptomatic screening and asymptomatic screening. Each would then branch into the eleven high-risk groups. The incremental cost effectiveness ratio (ICER) for each scenario was defined as the ratio of cost per screening (in MYR) to the TB cases detected.

$$\mathrm{ICER}=\frac{\Delta Cost}{\Delta Outcome}$$

$$\mathrm{ICER}=\frac{{\mathrm{Cost}}_{B}-{\mathrm{Cost}}_{A}}{{\mathrm{Outcome}}_{B}-{\mathrm{Outcome}}_{A}}$$

where

**Table 4:** Cost-Effectiveness Analysis of Different TB Screening Approach.

	Existing Approach§	Symptomatic Approach	Incremental
Cost Per Screening (MYR)	42.04	56.65	14.61
TB Cases Detected (per 1,000 Screening)	7.1	41.5	34.4
Cost-Effectiveness (Cost per TB Case Detected) (MYR)	5,932.78	1,363.62	–
ICER (MYR)	–	424.71	–

ICER incremental cost-effectiveness ratio.

§ Existing approach is defined as current TB screening approach, of which both symptomatic and asymptomatic high-risk groups were screened.

A is existing approach;

B is symptomatic TB screening

The decision tree model for cost-effectiveness analysis was based on the assumption that each screening procedure was conducted following similar procedure. Hence, eliminating the variation in terms of resources used such as personnel, machineries and consumables. There was also no variation of cost and resources used by different facilities. The model also assumed that each screening procedure strictly follows the TB screening guideline by MOH [12].

### 2.4 Sensitivity analysis

The uncertainty of costs, number of TB cases and probabilities data were investigated using Deterministic sensitivity analysis (DSA) and Probabilistic Sensitivity Analysis (PSA). In DSA, changes of ICER value were observed with various range of data. This would allow the robustness of the model to be explored. A set of minimum and maximum values were identified and introduced into the DSA. The outcome of DSA was depicted as Tornado Diagram, of which key drivers for the ICER can be determined.

Whereas, PSA used Bayesian method to assess the model outcome [24], [25]. To allow variation of data, a suitable statistical distribution was assigned (Table 2, Table 3, Table 4). Monte Carlo of 1,000 simulations were executed. The results are shown using Cost-effectiveness plane scatter diagram and Cost-Effectiveness Acceptability Curve (CEAC) allowing the cost-effectiveness level to be depicted at various level of WTP.

### 2.5 Budget impact analysis

A BIA was performed using Microsoft Excel. It is to estimate the expenditures and outcome of TB screening approach from provider (MOH) perspective over the span of 5 years, from 2018 to 2022. The BIA measure the direct costs of screening for the two strategies; existing TB screening approach, while the second was the symptomatic approach to TB screening. The estimated total number of TB screening for year 2018 was set as the population to receive TB screening. Data obtained from TBIS 204S revealed the total number of TB screening done in 2018 was 322,841 screening, for all the eleven high risk groups. The cost input for BIA was the cost per screening for each approach, estimated from the CEA. Meanwhile the outcome measured was the number of TB case detected. The incremental (or decremental) effect on cost (cost saving) and event (undetected or missed TB cases) were also included in the BIA. In order to measure the impact of actual monetary value (MYR) at each point in time, the cost used was not discounted [26].

## 3 Results

### 3.1 Cost-Effectiveness

**Fig. 2:**
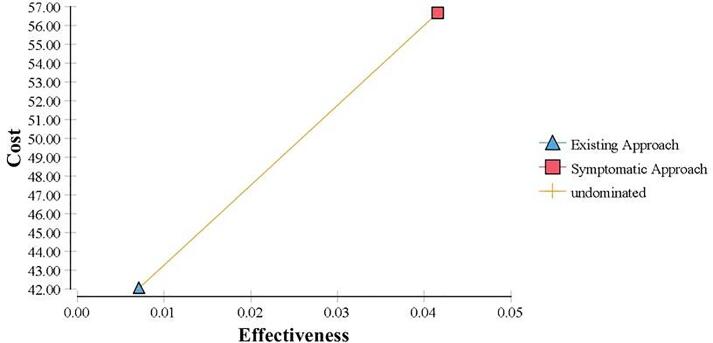
Cost-effectiveness Plane of symptomatic approach compared to the existing approach.

The result of CEA is shown in Table 4. The cost-effective plane in Fig. 2 depicts the cost-effectiveness comparing both strategies. Existing approach of TB screening had a lower cost per screening (MYR 42.04) compared to symptomatic approach (MYR 56.65). However, the cost-effectiveness (CE) value for the existing approach was more expensive (MYR 5,932.78 per TB case detected) compared to symptomatic approach (MYR 1,363.62 per TB case detected).

Switching from existing approach to the symptomatic approach would result in an increment of cost per screening by MYR 14.61 and TB case detection by 34.4 per 1,000 screening. Hence, resulting in an ICER of MYR 424.71 (Fig. 2). Symptomatic approach to TB screening was a strategy with much better outcome even though with a higher cost per screening compared to the existing approach.

### 3.2 Sensitivity analysis

**Fig. 3:**
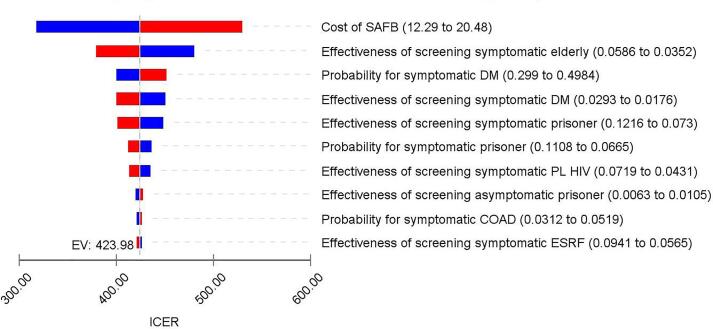
Deterministic Sensitivity Analysis for the symptomatic approach to TB screening versus the existing TB screening approach.

Fig. 3 shows the results of DSA for the existing approach versus the symptomatic approach. Despite changes made to the cost, probability and effectiveness data, the ICER value did not falls below zero. This indicates that the symptomatic approach would remain relatively dominant compared to existing approach. The costs of conducting SAFB was found as the key driver in determining the ICER value. Higher the cost of conducting SAFB would results in higher ICER value, while lower SAFB cost would results lower ICER.

**Fig 4:**
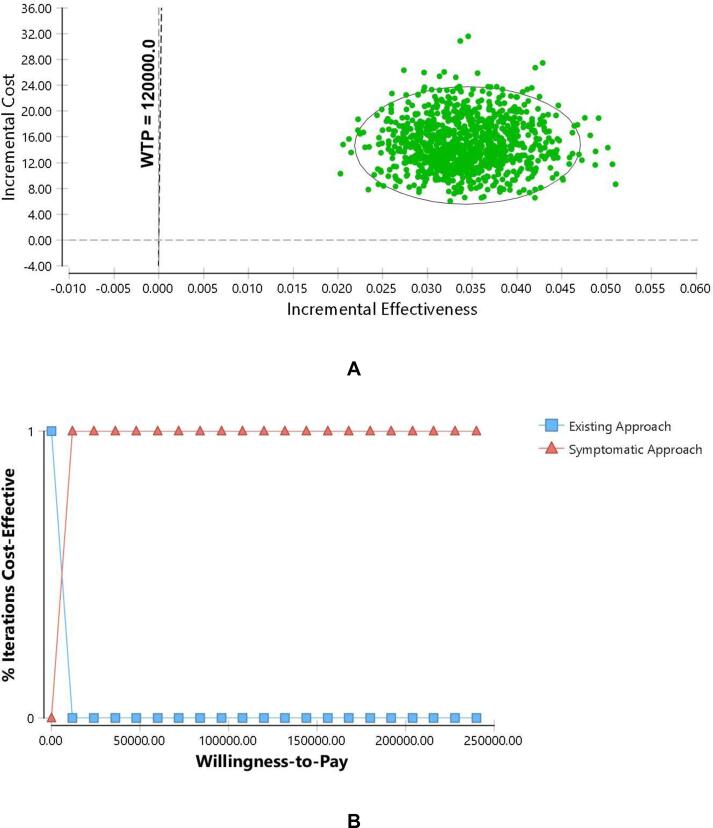
Probabilistic Sensitivity Analysis for the symptomatic approach to TB screening versus the existing TB screening approach; (A) Scatter plot of incremental cost and incremental effectiveness; (B) Cost-Effectiveness Acceptability Curve.

Fig. 4 depicts the PSA results of symptomatic approach versus existing approach. The cost-effectiveness plane shows the result of 1,000 Monte Carlo simulations. It is presented as scatter plot in a graph form with y-axis represents of incremental cost (per TB screening), while the x-axis represents incremental effectiveness (number of TB cases detected). Generally, the symptomatic approach was showed to be more expensive and more effective compared to the existing approach, with the plot mostly occupied the northeast area of the graph. The CEAC demonstrates that 100 % of the iterations resulted in symptomatic approach being more cost-effective of the two strategies almost throughout of all WTP values, up to MYR 240,000.

### 3.3 Budget impact analysis

**Fig. 5:**
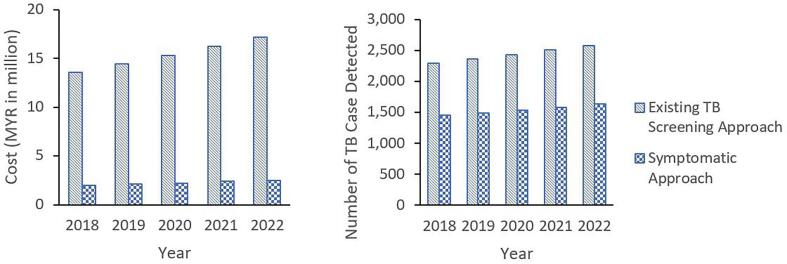
Estimated budget impact from years 2018 to 2022 for TB screening strategies. The estimated budget impact was based on number of total screening done for the whole country in the year 2018 based on TBIS 204S.

Table 5 shows five years BIA for different TB screening strategies from the year 2018 to 2022. Existing TB screening approach will result in estimated cost of MYR 13.6 million to 17.2 million yearly, while symptomatic approach to TB screening would costs around of MYR 2.0 to 2.5 million yearly (Fig. 5). In total, existing TB screening approach will result in 1,714,007 screening throughout the 5 years, which will cost an estimated total of MYR 76.6 million. This strategy results in an estimated total of 12,169 TB case detected over five years period. Whereas, symptomatic approach to TB screening will result in total of 185,456 screening over five years, at the same time costs around MYR 11.2 million over the same period. However, this strategy only results in a total detection of total 7,696 TB case. Thus, switching from the existing strategy to the screening approach focusing on the symptomatic will result in costs saving as much as MYR 65.5 million. However, this will also result in 4,473 TB cases not being detected (TB cases missed).

**Table 5:** Budget Impact Analysis of Different TB Screening Strategies.

	2018 (base)	2019	2020	2021	2022	Total for Last 5 Years
Number of TB Screening§	322,841	332,526	342,502	352,777	363,361	1,714,007
Existing TB screening approach						
Cost per screening (MYR)	42.04	43.30	44.60	45.94	47.32	–
Number of Symptomatic¥	34,931	35,979	37,059	38,170	39,316	185,456
Number of Asymptomaticɸ	287,910	296,547	305,443	314,607	324,045	1,528,552
Total number of screening	322,841	332,526	342,502	352,777	363,361	1,714,007
Total Cost (MYR in million)	13.6	14.4	15.3	16.2	17.2	76.6
Number of TB case Detected¶	2,292	2,361	2,432	2,505	2,580	12,169
Symptomatic Approach						
Cost per screening (MYR)	56.65	58.35	60.10	61.90	63.76	–
Number of symptomatic	34,931	35,979	37,059	38,170	39,316	185,456
Total Cost (MYR in million)	2.0	2.1	2.2	2.4	2.5	11.2
Number of TB case DetectedŦ	1,450	1,493	1,538	1,584	1,632	7,696
Cost Saving (MYR in million)	11.6	12.3	13.0	13.8	14.7	65.5
TB cases undetected (missed)	843	868	894	921	948	4,473

§ Total number of screening done for the 11 high-risk groups in TB for Malaysia. Data was based on total number of TB screening in TBIS 204S for the whole country.

¥ 10.82% was estimated from Cost-Effectiveness Analysis. Refer to Table 2.

ɸ The number of asymptomatic was calculated by deducting the total number of screening with the number of symptomatic.

¶ 7.1 TB case detected per 1000 screening from Table 4.

Ŧ 41.5 TB case detected per 1000 screening from Table 4 Inflation rate of 3.0 % was used.

## 4 Discussion

The results showed that the symptomatic approach was more cost-effective compared to the existing approach in terms of high-risk groups TB screening. This is in concordance with the past studies showing that TB case detection can be improved through symptomatic screening such as eliciting the history of cough among the patients [7]. On average, the existing approach would cost around MYR 5,932.78 to detect one TB case. For comparison, the cost was significantly lower through the symptomatic approach with around MYR 1,363.62 per TB case detected. Hence, it would be much more expensive screening both the asymptomatic and asymptomatic to get a similar outcome. This study also found that ICER value was greatly affected by the cost of conducting SAFB. As the cost of conducting sputum AFB becomes more expensive, the ICER value would become much higher, and vice versa. Therefore, to keep the ICER lower, the cost of conducting SAFB must be kept as low as possible.

Prior studies have discussed the presence of TB symptoms (such as productive cough, chest pain, shortness of breath, haemoptysis, fever, night sweats, and weight loss) among high-risk groups. Nonetheless, high-risk groups with TB such as the PL HIV hardly presented with any typical symptom, while some even presented with unspecific symptoms. Moreover, recent studies also found that a small proportion of these high-risk groups presented with minimal symptoms or asymptomatic, especially those living in countries with a high disease burden of TB [27]. For example, CXR for HIV patients infected with TB is less likely to showed cavitation compared to the TB patient not infected with HIV. In addition, about 22 % of HIV patients infected with TB showed to have normal CXR findings [28]. Despite that, many reports and studies suggested the inclusion of systematic screening as part of the screening procedure for the TB screening programme [17], [29]. The presence of TB symptoms would further improve the detection of TB through screening methods.

In CEA, the decision of whether one strategy is better or worse compared to the alternative greatly depends on the ICER. Switching from the existing approach to the symptomatic approach of TB screening will result in higher cost but also higher outcomes. The ICER value of MYR 424.71 reflects that additional one TB case detection requires an additional cost of MYR 424.72 by switching from the existing approach to the symptomatic approach. This value is much lower compared to the WTP threshold of MYR 120,000, and thus, suggests the symptomatic approach is cost-effective. Since the number of symptomatic cases affects the number of screening done, therefore, the proportion of active TB cases presented with TB symptoms are very crucial in deciding the strategy worth in terms of the value and outcome to the provider. Higher the number of cases presented with a symptom would result in more cost-effectiveness of the screening programme.

As much as the result of this study supports the implementation of a symptomatic approach to TB screening, it seems to contradict prior studies, which suggested that the undiagnosed TB patients in the community have a lack of typical symptoms and majorities being asymptomatic. This might indirectly influence the outcome of the TB screening programme, resulting in much lower TB case detection [30]. Consequently, even though it will be a cost-saving option to implement a TB screening only among the symptomatic, this will also result in some active TB cases being undetected in the community due to the low symptomatic yield among the community. Hence, unnecessary neglect of this group of people would result in difficulty in controlling the spread of TB in the community.

The BIA suggested that switching to a symptomatic approach to TB screening would result in an estimated total cost-saving as much as MYR 65.5 million over the five years. This also means that about 4473 TB cases will be undetected over the same period. As much as CEA is concerned, switching from an existing approach to the symptomatic approach would have opposite results. This, however, assuming that the number of screening of symptomatic approaches can match those of the existing strategy. Due to the low symptomatic yield among the high-risk groups, it is impossible to match those numbers, unless there are other methods or algorithms available that can increase the yield of symptomatic approach to TB screening. This can either be achieved by modifying or loosening the criteria for TB symptom definition or creating a new algorithm for symptomatic TB detection [17]. While not all high-risk groups need a full-blown TB symptom, some might need a much milder TB symptom. The conflicting results of CEA and BIA showed in this study are often noticeable in other economic evaluation studies as well, especially in real practice [31]. Therefore, both measures need to be taken into consideration before any decision is made.

The main strength of this study is the inclusion of diverse high-risk groups in the analysis. Hence, providing the overall view on TB screening strategies and at the same time can be used for comparison purposes. The inclusion of BIA as a supplement to the CEA allows the projection of cost and the consequence for five years as well as acts as a valuable decision guide for the policymakers [31]. This will provide a greater view of the impact on budget and outcome throughout five years period. For policymakers, it grants them the leverage in making the decision not only based on the cost-effectiveness measures but also based on the real impact towards the programme if such strategy is implemented [32]. In addition, this study also incorporated input from the MOH, who is behind the TB screening programme in Malaysia. This input is crucial in translating the analysis to local settings.

The policymaker can use the results of this study in deciding to improve the TB screening programme. While TB screening is known as the foundation in TB elimination programme, the budget constraints received for health programme requires policymakers to re-think the strategy for TB screening programme [33]. At the upper management level, the BIA provides the policymakers the estimates on costs incurred as well as the benefit gained or lost by adopting either of the strategy [31], [32]. Hence, it depends on the policymakers to decides which strategy is suitable based on the budget availability.

National Strategic Plan for Tuberculosis Control for Malaysia (2016–2020) has outlined few targets to be achieved by the year 2020. One of those is to achieve a TB notification rate (all cases) of 100 per 100,000 population [34]. To achieve this, a symptomatic approach to TB screening alone is not adequate, of which according to the BIA would only result in detection of 1450 to 1632 TB cases (which is around 5 per 100,000 population using the estimated population of 32.7 million in 2020 by Department of Statistic Malaysia) [35]. Hence, a carefully planned strategy needs to be put in place to achieve this target. While it is not a misguided practice to implement a symptomatic approach to TB screening and use the remaining cost-saving to patch up the TB programme with other proven practices such as latent TB screening and treatment, there is no hard blanket that can fit all circumstances.

Notwithstanding the above, this study is not without limitations. Firstly, this study used the number of TB cases detected as a measure of effectiveness rather than the most common measures such as Disability Adjusted Life Year (DALY) and Quality Adjusted Life Year (QALY) used by similar studies. Hence, not suitable for comparison purposes. Nonetheless, few studies used the number of TB cases detected as their effectiveness measure [7], [19], [36]. Using general effectiveness measures such as TB cases detected may not reflect the true burden of the disease. Hence, it may underestimate or overestimate the cost-effectiveness value since an elderly and young adult would have different QALY and DALY values. Furthermore, since this study only used TB data from Sabah and Sarawak, the results might not be representative of the TB burden in the country. Research in the future should aim at the modelling of TB screening programme by taking into account various factors and systematic screening. In addition, research on screening and management of latent TB is also suggested to complement the result of this study.

## 5 Conclusions

In conclusion, policymaker should weigh the inevitable trade-off between saving costs and achieving their goals in the national TB control programme to increase TB case detection. While screening only the symptomatic will results in more than half of TB cases detected, the subsequent marginal number of TB case detection will be much more expensive for the policymakers. A cost-effectiveness study might help in providing a piece of hard evidence but the results of BIA should also be taken into consideration in projecting the impact of such strategy, in the long run, making sure that the decision is in line with the objectives of national TB programme in eliminating TB.

## Availability of data and materials

All data generated or analysed during this study are included in this published article.

## Consent for publication

Not applicable.

## Ethical statement

This study was conducted according to the guidelines of declaration of Helsinki and did not include any identifiable human data. This study was registered under National Medical Research Register, MOH Malaysia (NMRR-19-3443-51729) and has attained ethics approval from Medical Research and Ethics Committee (MREC), MOH Malaysia. This study used secondary data analysis and had received informed written consent from Disease Control Division, MOH as well as Sarawak and Sabah State Health Department.

## CRediT authorship contribution statement

**Nor Zam Azihan Mohd Hassan:** Conceptualization, Methodology, Project administration, Data curation, Formal analysis, Software, Writing – original draft, Visualization. **Asmah Razali:** Methodology, Investigation, Writing – review & editing, Validation. **Mohd Shaiful Jefri Mohd Nur Sham Kunusagaran:** Data curation, Investigation, Writing – review & editing, Validation. **Farhana Aminuddin:** Writing – review & editing, Validation.

## Declaration of Competing Interest

The authors declare that they have no known competing financial interests or personal relationships that could have appeared to influence the work reported in this paper.
